# Identifying profiles of service users in housing services and exploring their quality of life and care needs

**DOI:** 10.1186/s12888-016-1122-0

**Published:** 2016-11-23

**Authors:** Neis A. Bitter, Diana P. K. Roeg, Chijs van Nieuwenhuizen, Jaap van Weeghel

**Affiliations:** 1 Department of Social and Behavioral Sciences, Tranzo Scientific Center for Care and Welfare, Tilburg University, PO Box 90153, 5000 LE Tilburg, The Netherlands; 2 GGzE Centre for Mental Health Care, PO BOX 909, 5600 AX Eindhoven, The Netherlands; 3 Phrenos Centre of Expertise, PO Box 1203, 3500 BE Utrecht, The Netherlands; 4 Parnassia Group, Dijk en Duin Mental Health Centre, PO Box 305, 1900 AH Castricum, The Netherlands

**Keywords:** Recovery, Rehabilitation, Housing services, Quality of life, Care needs, Severe mental illness

## Abstract

**Background:**

Housing services aim to support people with mental illness in their daily life and recovery. As the level of recovery differs between service users, the quality of life and care needs also might vary. However, the type and amount of care and support that service users receive do not always match their recovery. In order to improve the quality of care, this study aims to explore whether subgroups of service users exist based on three dimensions of recovery and to examine and compare the quality of life and care needs of the persons in these subgroups.

**Methods:**

Latent class analysis was performed with data from 263 service users of housing services in the Netherlands. Classes were based on three variables: personal recovery (Mental Health Recovery Measure), social recovery (Social Functioning Scale), and clinical recovery (Brief Symptom Inventory). Subsequently, the quality of life (MANSA) and care needs (CANSAS) of the different classes were analysed by the use of descriptive and inferential statistics.

**Results:**

Three classes could be distinguished. Class 1 (45%) comprised of people who score the highest of the three classes in terms of personal and social recovery and who experience the least number of symptoms. People in class 2 (44%) and class 3 (11%) score significantly lower on personal and social recovery, and they experience significantly more symptoms compared to class 1. The distinction between class 2 and 3 can be made on the significantly higher number of symptoms in class 3. All three classes differ significantly on quality of life and unmet needs.

**Conclusions:**

The quality of life of service users of housing services needs improvement, as even persons in the best-recovered subgroup have a lower quality of life than the average population. Workers of housing services need to be aware of the recovery of a client and what his or her individual needs and goals are. Furthermore, better care (allocation) concerning mental and physical health and rehabilitation is needed. Care should be provided on all dimensions of recovery at the same time, therefore mental health care organisations should work together and integrate their services.

**Trial registration:**

ISRCTN registry ISRCTN77355880 retrospectively registered 05/07/2013.

## Background

Since the mid-twentieth century the importance of long-term mental health care in a hospital setting has lost ground in the Western world. Influenced by national policies, traditions and resources, different countries have gone through different processes of deinstitutionalisation [1, 2]. This has led to a broad range of services characterised by a strong emphasis on community mental health care and an increase of housing services for people with severe mental illness (SMI) [3–7]. These services support service users in their daily lives and aim to support them in their recovery. In practice, their support mostly addresses practical daily care and nursing, but also assists the service users with engaging in meaningful daily activities and societal participation [8, 9]. Nevertheless, these people still report several unmet needs [10]. According to Slade et al. [11] mental health needs ‘include broad domains of health and social functioning, which are necessary to survive and prosper in the community’. The fulfilment of needs is related to a person’s quality of life, as quality of life is a result of a persons degree of satisfaction with major life domains [12]. In several studies, unmet needs appeared to be associated with a lower quality of life [11, 13–16]. Furthermore, the societal participation of service users is limited. For example, 10–20% have regular employment and 40% have no paid or voluntary work at all [17, 18]. Hence, we can conclude that housing services can still improve the quality of care for their service users.

Previous studies have demonstrated that the type and amount of care and support that service users receive do not always match their recovery. For example, service users who live in staffed sheltered facilities have comparable levels of functioning and problem severity compared to service users receiving outpatient housing support [17, 19]. This raises the questions of to what extent housing services provide the appropriate support to their users and to what extent the recovery needs of these service users are met. An earlier study [20] showed that service users of housing services experienced the most unmet needs with respect to mental and physical health and social contacts. This study also showed that workers and service users have different perspectives on unmet needs. Needs concerning social contacts and meaning in life appeared to be less frequently reported in treatment plans than were needs concerning self-care. Apparently, a discrepancy exists between the experienced needs of service users and the actual support provided. The present study, therefore, focuses on the needs and quality of life of these service users and to what extent these are related to their recovery.

Housing services aim to provide ‘recovery oriented care’. Several experts have described that recovery contains multiple dimensions, both objective and subjective [21–24]. An example of a classification that is often used in the Netherlands is the trichotomy – clinical, social and personal recovery [23, 25]. Clinical recovery refers to a decrease in clinical symptoms such as hallucinations, anxiety or depressive feelings [26]. Social recovery is about regaining everyday functioning, for example in work, social relationships, housing and family life [27]. Personal recovery refers to a person’s own experience of his/her recovery; it is about hope, empowerment, self-determination and regaining the identity of someone who is living a meaningful life despite the presence of symptoms [21, 28, 29]. A recovery process is very personal and can fluctuate [30] and the dimensions influence each other constantly. Therefore, treatment and support for people with SMI mental illness should focus on all three dimensions of recovery [25], and should be centred around their individual needs and quality of life [31].

In order to improve the quality and focus of support of community-based services, it is important to gain a better understanding of the recovery of their users, their corresponding needs and perceived quality of life. Therefore, the aim of this study is to explore whether subgroups exist based on three dimensions of recovery (clinical, personal and social), as well as to examine the quality of life and care needs within and between these subgroups.

## Methods

### Procedure

This study was part of a clinical trial on the effectiveness of the Comprehensive Approach to Rehabilitation (CARe) Methodology, which is being executed in 14 teams selected from three organisations for housing services in the Netherlands [32]. In the Netherlands, practical support on the field of daily living and participation for people with SMI is often offered by housing services. They do not provide the service users’ medical and psychiatric treatment. Most service users receive treatment from multidisciplinary community treatment teams from local mental health care organisations. Housing services offer several forms of housing. Sheltered housing is a residential facility with 24-h supervision. Supported independent living is a service for people who live on their own and receive just a couple of hours of support per week for certain domains. The participating teams all provide sheltered housing and/or supported independent living services. To inform service users about the study, an information meeting at each facility was organised and all service users received an information brochure. Subsequently, service users were approached individually by the researcher or via the staff to take part in an interview. Beforehand participants were asked to sign an informed consent to take part in the study and to permit use of their information. Each participant received information about his or her right to withdraw from the study at any time. The study received ethical approval from the Medical Research Ethics Committee of the Elisabeth Hospital in Tilburg (NL41169.008.12). The trial registration number is ISRCTN77355880 (http://www.isrctn.com/ISRCTN77355880).

### Participants

Participants were recruited between September 2012 and April 2013 in 14 teams providing services to 631 people (all 18 years and older). Exclusion criteria for the study were: too little knowledge of the Dutch language to fill out the questionnaire and/or being unable to give informed consent due to cognitive impairment or clinical symptoms. In total, 263 people agreed to participate and met the inclusion criteria. Participants and non-participants did not differ significantly on gender, age and diagnosis.

### Measures

Measures were chosen that met the aims of recovery-oriented care, were subjective and client-rated in nature and had good psychometric properties.

- **Personal recovery** was measured using the Dutch version of the Mental Health Recovery Measure (MHRM), an instrument developed to assess the recovery process [33]. The MRHM is a self-report instrument with 30 items. The Dutch version is comprised of three subscales: ‘self-empowerment’ (α = 0.90), ‘learning and new potentials’ (α = 0.86) and ‘spirituality’ (α = 0.94) [33]. All items are rated using a five-point Likert scale that ranges from ‘*strongly disagree*’ to ‘*strongly agree*’.
- The Social Functioning Scale (SFS) was used to measure **social recovery**. The client-rated scale (α = 0.80) consists of 19 items and four checklists on seven domains: social engagement/withdrawal, interpersonal behaviour, pro-social activities, recreation, independence-competence, independence-performance and employment/occupation [34].
- **Clinical recovery** was measured by use of the Brief Symptom Inventory (BSI) [35, 36]. This is a 53-item self-report questionnaire (α = 0.96). This instrument assesses clinical symptoms during the past week. The items are rated using a five-point scale (0–4), ranging from ‘*not at all*’ to ‘*extremely*’. The BSI has nine subscales: somatisation, obsessive-compulsive, interpersonal sensitivity, depression, anxiety, hostility, phobia, paranoia and psychoticism. The total of all items is calculated as a total score of psychological functioning [35].
- **Quality of life** was assessed using the Manchester Short Appraisal (MANSA), an instrument to measure quality of life in people with mental illness. The MANSA (α = 0.74) consists of 12 subjective items with a seven-point Likert scale (‘*could not be worse*’ – ‘*could not be better*’). Besides the subjective questions on satisfaction, the MANSA contains four yes/no questions, for example, about the presence of a good friend [37, 38].
- **Need for care** was measured using a 27-item client-rated version of the Camberwell Assessment of Needs Short Appraisal Schedule (CANSAS). With this instrument, the service user can score a health or social need as ‘*no need*’, ‘*met need*’ or ‘*unmet need*’ [39].
- The following **demographic variables** were collected: age, gender, marital status, employment status and living situation. These demographics were measured by use of a client-rated form developed for the study. The key workers were asked to fill out a form with questions about the diagnosis and care use of the service user.

### Analysis

Latent Class Analysis (LCA) [40] was used to identify subgroups of service users based on three critical dimensions of recovery: personal recovery, social recovery and clinical recovery. These dimensions were operationalised by, respectively: MHRM (measuring personal recovery), SFS (measuring social functioning) and BSI (measuring clinical symptoms). LCA is a statistical and probabilistic, method that can be used to classify individuals from a heterogeneous population into smaller more homogenous unobserved subgroups.

The analysis consisted of two steps. The first step was determining the number of classes based on the three dimensions of recovery. Model fit indices were used to select the model with the most suitable number of clusters. The Bayesian Information Criterion (BIC) and the Aikake Information Criterion 3 (AIC3) were used for this purpose. These measures provide information about the relative quality and the parsimony of a statistic model. The BIC and AIC have the lowest values on the best model [41, 42]. Furthermore, the classification error was taken into account; this value represents the chance that a participant is assigned to the wrong class. Finally, we looked at the bivariate residuals. These should be < 4, as bivariate residuals > 4 imply a possible correlation between the included variables. The LCA was conducted with Latent Gold [43].

The aim of the second step was to map the classes in terms of care needs and quality of life and demographics. Furthermore, the extent to which the classes differ significantly on these variables was tested. For continuous variables, analysis of variance (ANOVA) was used. For categorical variables, chi-square tests were used. A *p*-value < 0.05 was used to indicate statistical significance. These analyses were executed with SPSS 19.0. Furthermore, effect sizes (Eta squared for ANOVA and Cramer ‘s V for the chi-square tests), were calculated and reported.

## Results

### Results of the LCA

We compared the fit indices of the models with one to seven clusters. Table 1 shows the results of this analysis. The three-cluster model was chosen as the most appropriate solution based on the clinical interpretation and the following criteria. The BIC (3229.9668) and the AIC3 (3178.5238) are the lowest for the three-cluster model. The classification error for this model is 0.1642, which is acceptable. Moreover, the bivariate residuals were below four.

**Table 1:** Result Latent Class Analysis (*N* = 263)

		LL	BIC (LL)	AIC3 (LL)	No of parameters	Class. Err.
Model 1	1 cluster	−1652.9335	3339.2999	3323.8670	6	0.0000
Model 2	2 cluster	−1579.0862	3230.6104	3197.1724	13	0.1194
Model 3*	3 cluster	−1559.2619	3229.9668	3178.5238	20	0.1642
Model 4	4 cluster	−1549.4542	3249.3565	3179.9084	27	0.2164
Model 5	5 cluster	−1541.5716	3272.5965	3185.1433	34	0.2036
Model 6	6 cluster	−1532.2652	3292.9887	3187.5303	41	0.2012
Model 7	7 cluster	−1526.4417	3320.3468	3196.8834	48	0.1932

*The selected model

### Class descriptions

The mean age of the whole group of participants was 50; 65% of them were male (Table 2). At 51%, psychotic disorder was the most reported diagnosis. A total of 72.5% of the participants lived in a supported housing facility and 27.5% received supported independent living services. Concerning the demographics (Table 3), no significant differences were found between the classes (age, having a partner, living situation, work situation, diagnosis and amount of contact with workers), with the exception of gender. Class 3 contains a higher percentage of women (66%; *p* < .001) than do the other classes (29% in class 1 and 34 % in class 2).

**Table 2:** Descriptive variables per class (*N* = 263)

	Whole sample N = 263	Class 1 n = 118 (45%)	Class 2 n = 116 (44%)	Class 3 n = 29 (11%)	*p*
Age (mean ± SD)	50.16 (13.85)	48.22 (13.38)	51.44 (13.47)	52.93 (16.44)	ns
Gender					
(Male)	170 (65%)	84 (71%)	76 (66%)	10 (34%)	<.001
Female	93 (35%)	34 (29%)	40 (34%)	19 (66%)	
Partner					ns
Yes	37 (14%)	12 (10%)	19 (16%)	6 (21%)	
No	226 (86%)	106 (90%)	97 (84%)	23 (79%)	
Living situation					ns
Supported housing	190 (72.5%)	92 (78%)	79 (68%)	19 (68%)	
Supported independent living	72 (27.5%)	26 (22%)	37 (32%)	9 (32%)	
Work					ns
Paid work	9 (3%)	6 (5%)	1 (1%)	2 (7%)	
Sheltered work	23 (8%)	15 (13%)	6 (5%)	2 (7%)	
No work	148 (56%)	56 (48%)	74 (64%)	18 (62%)	
Unpaid work	64 (24%)	34 (29%)	26 (29%)	4 (14%)	
Retired	14 (5%)	5 (6%)	7 (6%)	2 (7%)	
Diagnosis					ns
Psychotic disorder	124 (51%)	59 (57%)	51 (47%)	14 (50%)	
Mood disorder	23 (10%)	8 (8%)	13 (12%)	2 (7%)	
Anxiety disorder	10 (4%)	3 (3%)	5 (5%)	2 (7%)	
Autism spectrum disorder	18 (7%)	8 (8%)	6 (6%)	4 (14%)	
Personality disorder	23 (10%)	7 (7%)	12 (11%)	4 (14%)	
Substance use disorder	12 (5%)	3 (3%)	8 (7%)	1 (4%)	
Other/none	6 (2%)	3 (3%)	1 (1%)	1 (4%)	
Contact with housing service					ns
≥Once a day	144 (60%)	59 (56%)	68 (63%)	17 (63%)	
>Once a week	55 (23%)	29 (28%)	20 (19%)	6 (22%)	
Once a week	31 (13)%	14 (13%)	13 (12%)	4 (15%)	
<Once a week	10 (4)%	3 (3%)	7 (7%)	0 (0%)

**Table 3:** Scores on measures per class and significant differences between the classes (*N* = 263)

	Whole sample *N* = 263	Class 1 *n* = 118 (45%)	Class 2 *n* = 116 (44%)	Class 3 *n* = 29 (11%)	Eta square	*p*
Included in the LCA						
SFS (N = 263)	111.05 (24.1)	120.4 (21.0)^a^	105.51 (23.9)^b^	95.14 (22.1)^b^	0.14	<.001
MHRM (N = 262)	3.48 (0.52)	3.85 (0.39)^a^	3.21 (0.37)^b^	3.03 (0.53)^b^	0.42	<.001
BSI (N = 257)	0.76 (0.62)	0.32 (0.2)^a^	0.87 (0.32)^b^	2.1 (0.53)^c^	0.76	<.001
Included in post-hoc analysis						
MANSA	4.02 (0.69)	4.43 (0.52)^a^	3.74 (0.58)^b^	3.44 (0.74)^c^	0.31	<.001
CANSAS Unmet needs	4.16 (3.03)	2.81 ((2.21)^a^	4.85 (2.97)^b^	7.07 (3.37)^c^	0.22	<.001
CANSAS Met needs	8.12 (3.19)	8.44 (3.02)	7.88 (3.03)	7.71 (4.31)	-	ns
CANSAS No needs	14.55 (3.36)	15.56 (3.56)^a^	14.09 (2.81)^b^	12.14 (2.90)^c^	0.11	<.001

Classes with different characters (a, b, c) significantly differ on the indicated variable. *p* < .05; classes with similar characters do not differ from each other

Interpretation Eta squared: .02 = small; .13 = medium; .26 = large

Table 3 shows the mean scores of the three recovery measures for each class. Class 1 (45% of the respondents) represents service users who have the highest scores on social functioning (SFS = 120.4) and personal recovery (MHRM = 3.85) and the lowest scores on symptoms (BSI = 0.32). The service users in class 2 (44% of the respondents) score significantly lower on social functioning (SFS = 105.5) and personal recovery (MHRM = 3.21) than do the service users in class 1 and higher on symptoms (BSI = 0.87). The service users in class 3 (11% of the respondents) have the lowest scores on social functioning (SFS = 95.14) and personal recovery (MHRM = 3.03) and the highest scores on symptoms (BSI = 2.1). All three classes differ significantly (p < .001) on clinical symptoms (respondents in class 1 showing the fewest number of symptoms); this difference is also the strongest (eta squared = 0.76). Service users in class 1 differ significantly from users in classes 2 and 3 on all three dimensions of recovery (*p* < .001). Classes 2 and 3 differ significantly on symptoms (*P* < .001), and not on social functioning and personal recovery.

Compared with norm scores of the BSI, service users in class 1 (mean 0.32) have fewer clinical symptoms than do outpatients (norm score = 0.44–0.86) and slightly more than do non-patients (norm score = 0.15–0.29). Service users in classes 2 and 3 experience, respectively, a comparable number (mean 0.87) and more (mean 2.1) clinical symptoms than do outpatients [44].

### Care needs

With regard to the number of ‘met needs’ (needs for which a person receives care or support), no significant differences between the three groups were found. The average number of met needs is around eight in all classes. Concerning the number of ‘unmet needs’ and the number of ‘no needs’, significant differences exist between the three groups (*p* < .001) (Table 3). Service users in class 1 have the lowest average number of unmet needs (i.e. three). Service users in class 2 have five and users in class 3 have seven unmet needs. When comparing the groups on the percentage of service users (%) for whom a certain need is unmet (Table 4), the strongest differences exist in the needs with regard to ‘psychological distress’ (class 1: 11.3%, class 2: 39.4%, class 3: 78.6%) and ‘safety for self’ (class 1: 0.0 %, class 2: 7.3%, class 3: 35.7%). Furthermore, a strong difference is visible concerning the need ‘meaning and recovery’ (class 1: 19%, class 2: 42,3%, class 3: 71,4%).

**Table 4:** Percentage of service users for whom a certain need is unmet

Care need (CANSAS)	Whole sample *N* = 263%	Class 1 *n* = 118 (45%)%	Class 2 *n* = 116 (44 %)%	Class 3 *n* = 29 (11%)%	Cramer’s V	*P*
Accommodation	19.0	19.8	17.4	21.4	-	ns
Food	10.2	4.3	12.8	25.0	0.165	<.01
Household skills	5.1	2.6	5.5	14.3	0.143	<.05
Self-care	3.5	1.7	3.7	10.7	0.179	<.01
Daily activities	23.6	12.1	30.2	46.4	0.197	<.01
Physical health	32.4	20.0	41.1	50	0.197	<.01
Psychotic symptoms	9.6	4.4	10.1	28.6	0.182	<.01
Condition/treatment info	12.3	7.8	14.7	21.4	-	ns
Psychological distress	31.0	11.3	39.4	78.6	0.346	<.001
Safety to self	7.1	0.0	7.3	35.7	0.346	<.001
Safety to others	2.4	2.6	0.9	7.1	-	ns
Alcohol	4.7	1.7	8.3	3.6	-	ns
Drugs	2.0	1.7	1.9	3.6	-	ns
Company	31.6	19.7	38.0	57.1	0.192	<.01
Intimate relationships	24.3	21.4	26.9	21.4	-	ns
Sexual expression	19.5	15.8	23.8	18.5	-	ns
Child care	2.4	2.6	1.8	3.6	-	ns
Basic educations	6.7	8.6	3.7	10.7	-	ns
Telephone	5.1	5.1	3.7	10.7	-	ns
Transport	18.6	10.3	26.9	21.4	0.170	<.01
Money	21.3	12.8	28.4	28.6	0.150	<.05
Benefits	8.3	6.8	11.0	3.6	-	ns
Paid work	34.4	35.9	31.2	39.3	-	ns
Side effects medication	24.4	20.2	28.4	25.9	-	ns
Meaning and recovery	34.7	19.0	42.3	71.4	0.263	<.001
Judicial	3.1	2.6	2.8	7.1	-	ns
Sleep	22.5	12.1	28.4	42.9	0.194	<.01

Interpretation Cramer’s V: .15 = small; .25 = medium; .35 = large

There are also several needs that are frequently unmet (>20%) and for which there is no significant difference between the classes. These are: intimate relations (24.3% of whole sample), paid work (34.4% of whole sample) and side effects of medication (24.4% of whole sample).

Besides the differences and similarities between the classes, we also looked at the most frequently (>35%) unmet needs per class. In class 1, this was ‘paid work’ (35.9%). In class 2, this was the case for: ‘meaning and recovery’ (42.3%), ‘physical health’ (41.1%), ‘psychological distress’ (39.4%) and ‘company’ (38.0%). In class 3, the following needs were reported as unmet by more than 35% of the service users: ‘psychological distress’ (78.6%), ‘meaning & recovery’ (71.4%), ‘company’ (57.1%), ‘daily activities’ (46.4%), ‘sleep’ (42.9%), ‘paid work’ (39.3%), and ‘safety to self’ (35.7%).

### Quality of life

The scores on quality of life differ significantly between the three classes (class 1: mean 4.43, class 2: mean 3.74, class 3: mean 3.44; eta squared = 0.31; *p* < .001). When comparing the scores with norm scores, we see that service users of all classes have a lower mean score than the average population (norm score = 5.27). Service users in class 1 have comparable scores as people with SMI (norm score = 4.69); users in class 2 and 3 have lower mean scores than people with SMI.

On several specific domains, significant differences exist between the classes (Table 5). The differences are the strongest on the domains ‘mental health’ (eta squared =0.24; *p* < .001), ‘physical health’ (eta squared = 0.18; *p* < .001), ‘life as a whole’ (eta squared = 0.20; *p* < .001) and ‘job (when having a job)’ (eta squared = 0.21; *p* < .05).

**Table 5:** Mean scores per quality of life item

Mansa item	Whole sample *N* = 263	Class 1 *n* = 118 (45%)	Class 2 *n* = 116 (44%)	Class 3 *n* = 29 (11%)	Eta squared	*P*
Total score	4.02	4.43^a^	3.74^b^	3.44^c^	0.31	<.001
Life as a whole	4.60	5.30^a^	4.14^ab^	3.55^b^	0.21	<.001
Job (when having one)	5.60 (*n* = 47)	5.94^a^ (*n* = 32)	4.91^b^ (*n* = 11)	4.75^ab^ (*n* = 4)	0.20	<.05
No job	4.37 (*n* = 218)	4.57 (*n* = 87)	4.34 (*n* = 106)	3.80 (*n* = 25)	-	ns
Financial situation	4.36	4.81^a^	3.98^b^	4.0^ab^	0.06	<.01
Amount and quality of friends	4.79	5.24^a^	4.54^b^	3.97^b^	0.08	<.001
Leisure activities	5.00	5.53^a^	4.62^b^	4.41^b^	0.11	<.001
Housing	5.16	5.48^a^	4.90^b^	4.90^ab^	0.04	<.05
Personal safety	5.41	5.75^a^	5.29^b^	4.52^c^	0.10	<.001
People with whom the individual lives	4.82	5.13^a^	4.49^b^	4.79^ab^	0.04	<.05
Living alone	4.89	5.22	4.63	4.73	-	ns
Sex life	4.08	4.43^a^	3.77^b^	3.89^ab^	0.03	<.05
Relationship with family	4.79	5.13^a^	4.65^ab^	3.97^b^	0.05	<.01
Physical health	4.11	4.90^a^	3.56^b^	3.14^b^	0.18	<.001
Mental health	4.36	5.22^a^	3.83^b^	3.00^c^	0.24	<.001

Classes with different characters (a, b, c) significantly differ on the indicated variable. *p* < .05; classes with similar characters do not differ from each other

Interpretation Eta squared: .02 = small; .13 = medium; .26 = large

When looking at the average number of domains on which people in a class are at least ‘mostly satisfied’ (mean > 5), we see that for people in class 1, this is the case for ten domains (life as a whole, job, amount and quality of friends, leisure activities, housing, personal safety, people with whom the individual lives, living alone, relationship with family and mental health). People in class 2 have one domain on which the average score is 5 or higher (personal safety); people in class 3 have none (see Table 5).

Regarding the average number of domains on which people in all classes score lower than ‘mostly unsatisfied’ (mean <4), we see that in class 1, there is no domain for which this is the case. People in class 2 have four domains on which this is the case (physical health, mental health, financial situation and sex life). People in class 3 have an average score of < 4 on seven domains (mental health, physical health, life as a whole, no job, sex life, relationship with family, and amount and quality of friends).

## Discussion

This study aimed to explore whether subgroups of service users in housing services exist based on three recovery dimensions, and to examine and compare the quality of life and care needs in these subgroups. We identified three subgroups of service users, which differed significantly in terms of clinical recovery. The clinically most recovered subgroup (class 1) showed only minor symptomatology: just slightly more than non-patients. This group also differed significantly from the other two subgroups on personal and functional recovery and can therefore be labelled as the most recovered subgroup of the three. Classes 2 and 3 represent people who experience more symptoms; comparable and much higher compared to outpatients respectively, and they also score the lowest on personal recovery and social functioning. Persons in class 1 mainly experience difficulties in their social recovery; persons in class 2 seem to stay mainly behind both in their personal and social recovery, while persons in class 3 experience problems in all recovery areas.

Although persons in the best-recovered subgroup found in this study have a comparable number of symptoms (score on the BSI) as healthy people, their quality of life appears to be much lower. Their quality of life was the highest of the three subgroups found, though still comparable with outpatients in other studies. Persons in the other two subgroups have a lower quality of life than do outpatients [12]. As improving quality of life is a central aim of mental health care, it is important to look at the deeper causes and search for possibilities to increase service users’ wellness. We aimed to do this by analysing the different quality of life domains and unmet needs.

When looking at the total picture of unmet needs and quality of life on different domains, it becomes visible that the difference between the classes is mainly the number of domains on which a person needs support. The priority of service users in class 1 lays mainly on paid work and (intimate) relations. Service users in class 2 have these needs also, but in addition, they also need support regarding personal recovery and physical and mental health. Service users in class 3 experience similar problems as users in class 2; moreover they have more serious problems concerning their mental health.

Although the number of needs differ, it is remarkable that on some topics, notably paid work and intimate relations, the number of service users that experience an unmet need in these areas is comparable in all three classes. This indicates that persons suffering from severe psychological distress also have relevant needs in other areas such as work and relationships. It seems, therefore, to be unnecessary to wait until a person is recovered symptomatically to provide support in these areas. It may be possible, and recommendable, to provide support on all dimensions of recovery at the same time, guided by the individual life goals of the client. This corresponds to the growing insight that (vocational) rehabilitation has to be integrated in clinical services [45]. A successful example of this is the Individual Placement and Support model of supported employment, which is applied to support people in getting and maintaining competitive employment; a significant number of studies have proven that this is actually possible [46–49].

Another remarkable result from this study is that a small but distinct group (class 3, 11%) seems not to receive the psychiatric treatment they need or they may not profit enough from it. In this group, 79% have an unmet need concerning psychological distress, 71% on meaning and recovery, 50% on physical health and 36% have an unmet need concerning safety to oneself. It is worrisome that such a distinct group has so many serious unmet needs. More attention is needed in mental health care to support these people in their daily life and their recovery. Furthermore, in classes 1 and 2, also a high number of unmet needs were reported in the areas of physical and mental health. These also include the quality of life domains that people in classes 2 and 3 are often less satisfied with. This implies that service users of housing services may not receive enough mental and physical health care. This is, especially concerning physical health care, a well-known phenomenon [13, 50, 51]. It is also a complex problem, for which several explanations exist (e.g. lack of awareness, stigma and poor communication and cooperation between different care providers in the field of (mental) health care) [52–54].

Differences in demographics, living situations and amount of support are not evident between the three classes. Although class 1 seems to score high on the different dimensions of recovery, these people do not have paid work or live independently more often than do the people in class 3. Moreover, the number of people with paid work is very low (3%). It is remarkable that people who score high on personal recovery and experience few symptoms do not participate more in society than do people who are less recovered. This is in line with other studies; for example, De Heer-Wunderink et al. (2012) compared service users who lived independently with users who lived in a sheltered housing facility and found that their participation in social activities differed but that their vocational participation was similar. In addition, a study on Van Gestel et al.’s (2012) recovery profiles concluded that recovery could not be significantly related to work status. Furthermore, Valdes-Stauber (2015) found that the level of institutionalisation of service users of different housing services did not reflect the severity of their illness or functional impairments. In short, there seems to be a gap between clinical and personal recovery on the one hand, and participation in society on the other.

There are several possible explanations for this. First, housing services seem not to offer adequate support to service users concerning social inclusion; therefore, more effort should be given to this topic in these facilities [5, 14, 55]. Another possible explanation can lay in the often-impaired executive and cognitive functions in people with SMI, such as deficits in concentration, planning skills, self-regulation and motivation [56–58]. As a result, service users are not prepared to perform in, for example, the competitive labour market. Although there is a growing body of interventions that focus on cognitive rehabilitation [59–61], these interventions are still not broadly offered in (long-term) mental health care. Another explanation can be the impact of (self)stigma [62, 63]. It can be challenging for people recovering from a mental illness to become included in mainstream society, as stigmatisation of people with mental disorders is still widespread [64, 65]. Moreover, due to earlier disappointing experiences and the internalisation of stigma, people with SMI may lose self-esteem and self-efficacy. This in itself may lead to a decrease of initiative and motivation to participate in society, the so-called the ‘why try effect’ [66]. Lastly, people with SMI, and their relatives, can be uncertain about their possibilities and/or afraid of a relapse. As a consequence, they may have the tendency to avoid risks [67]. In sum, it is necessary to give more attention to rehabilitation and societal participation of all service users with SMI, regardless their recovery stage.

### Strengths and limitations

The strength of this study is that 263 service users with SMI participated. However, a limitation may be that the data used in this study were gathered in the context of another study [32]; therefore, the recruitment of participating organisations and service users was not totally random. When we compare these characteristics with (inter)national studies on service users of housing services, though, we can conclude that the participants of this study are representative for this target group [13, 19]. The use of validated client-rated measures is another strength. Due to this, we achieved insight in the actual state of how service users experience their recovery on several fields; even though housing services do not structurally collect this information in a validated and reliable way. This is, to our knowledge, the first study identifying and exploring recovery profiles of clients of housing services and their quality of life and care needs. This explorative approach offers new insights, which are not only relevant for housing services but also for other stakeholders in mental health care.

## Conclusions

Service users of housing facilities can be divided into three classes of recovery. Each class experiences a different level of quality of life and comes with a different type and number of unmet needs. It is important for workers of housing services to be aware of the recovery of a client and what his or her individual needs and goals are. One size does not fit all for service users of housing services. Nonetheless, similarities were also found. As service users in all classes have rehabilitation needs with regard to intimate relations and employment, attention for all dimensions of recovery at the same time is recommended. As it is important to provide care on al dimensions of recovery, it is necessary for mental health care organisations to work together and integrate their services to increase quality and continuity of care for people with long-term severe mental illness. Furthermore, more quantitative and qualitative research is needed to further explain the differences between the three groups in recovery, quality of life and care needs. This knowledge can be used to develop interventions or adjust the current practice in order to improve the quality of life.

## Abbreviations

AIC: Aikake Information Criterion
ANOVA: Analysis of Variance
BIC: Bayesion Information Criterion
BSI: Brief Symptom Inventory
CANSAS: Camberwell Assessment of Needs Short Appraisal Schedule
CARe: Comprehensive Approach to Rehabilitation
LCA: Latent Class Analysis
MANSA: Manchester Short Appraisal (MANSA)
MHRM: Mental Health Recovery Measures
SFS: Social Functioning Scale
SMI: Severe Mental Illness
